# Early Environments Shape Neuropeptide Function: The Case of Oxytocin and Vasopressin

**DOI:** 10.3389/fpsyg.2019.00581

**Published:** 2019-03-20

**Authors:** Adi Perry-Paldi, Gilad Hirschberger, Ruth Feldman, Orna Zagoory-Sharon, Shira Buchris Bazak, Tsachi Ein-Dor

**Affiliations:** Baruch Ivcher School of Psychology, Interdisciplinary Center Herzliya, Herzliya, Israel

**Keywords:** oxytocin, vasopressin, threat detection, early life stress, neuropeptidal function

## Abstract

Oxytocin (OT) and vasopressin (AVP) are neuropeptides that govern the social-emotional functioning of humans. We contend that to fully understand their function, research should consider how they are flexibly fitted to maximize survival and reproduction given the variety of human experience. In a series of two studies, we show that early life stress is associated with change in the core function of OT and AVP in evolutionary predictable ways: Under high early life stress, AVP promotes threat-detection capabilities, whereas OT motivates non-selective proximity seeking to others. Conversely, under low early life stress these neuropeptides have an opposite, yet adaptive response: AVP promotes low vigilance and preservation of energy, whereas OT increases detection of interpersonal flaws. Our results demonstrate the plasticity of neuropeptide functioning that mirrors the variance in human social-emotional functioning.

## Introduction

Since the discovery of neuropeptides (e.g., Smelik, 1960; De Wied, 1961), there has been a growing interest in understanding their various functions. Research has indicated that oxytocin (OT) and arginine vasopressin (AVP) oversee the social-emotional functioning of humans (e.g., social bonding, social aggression, vigilance to social threats; Bos et al., 2012), and the commonly held view is that these neuropeptides serve the same function among different people. Here we challenge this perspective and suggest that to fully understand the adaptive function of these neuropeptides, the plasticity of human behavior at times of threat must be considered. This plasticity is reflected in the interaction between the individual and his or her environment such that among individuals with a different life history, neuropeptides will be flexibly fitted to the specific environment (Taylor et al., 2006; Bartz et al., 2011; Olff et al., 2013), and will, thus, have different functions and promote different behaviors and cognitions. Accordingly, we present evidence showing that the function of OT and AVP changes in an evolutionary predictable way according to people’s early life stress – OT and AVP maximize alertness to threats and social-based survival for people who experienced high levels of early life stress, but not for those who experienced low levels of early life stress.

The plasticity of human behavior at times of threat was suggested by Boyce and Ellis (2005) who argued that we were perfected by evolution to be biologically sensitive to the environmental context, as well as by Belsky’s differential susceptibility theory (DST; Belsky, 1997, 2005). They suggested, for example, that developing heightened reactivity to stressors may confer selective advantages in certain social and ecological contexts. Specifically, heightened reactivity to stressors “increases adaptive competence in highly stressful environments by augmenting vigilance to threats and dangers and in highly protective environments by increasing susceptibility to social resources and ambient support.” (Boyce and Ellis, 2005, p. 272). In recent years, differential susceptibility models have expanded to include multistage developmental processes in which genetic variation interacts with exposure to early environmental factors, such as prenatal stress hormones, rearing and parental care (e.g., Belsky et al., 2009; Ellis et al., 2011; Pluess and Belsky, 2011; Belsky and Pluess, 2013). In a recent simulation study, Del Giudice (2016) has shown that these processes are plausible, have the potential to generate remarkably complex patterns of interplay between genotypes, phenotypes, and environments, and are compatible with evidence from twin research.

Research in attachment theory (Bowlby, 1982) also lend abundant evidence for the effect of early life stress on people’s cognitions, emotions and behaviors that last “from the cradle to the grave.” When children experience their caregivers as responsive and supportive, they develop a sense of attachment security, along with constructive strategies (e.g., support-seeking) for coping with threats and regulating emotions. Conversely, when caregivers are perceived as unavailable or unreliable, a child tends to develop an insecure attachment orientation marked by either attachment-system deactivating strategies for regulating emotions and social behavior (avoidant attachment) or attachment-system hyperactivating strategies (attachment anxiety) (see Mikulincer and Shaver, 2007; Cassidy and Shaver, 2008, for reviews). Recently, research has shown that these attachment styles, which were developed in response to different levels of early life stress, are linked with epigenetic changes in stress response (glucocorticoid receptor gene) and social tendencies (oxytocin receptor gene) (e.g., Ein-Dor et al., 2018). In the current research, we propose that early life stress would affect the function of OT and AVP in an evolutionary predictable way.

Research has indicated that OT governs social bonding and social cognition such as increased romantic connectedness (Schneiderman et al., 2012), provision of care to close others and the promotion of calming behaviors (Olff et al., 2013), especially in the presence of familiar people. Among people with a history of secure interactions and a relatively safe environment, OT optimizes the selection of one’s social network. Based on an accurate detection of social cues (Bartz et al., 2010), OT promotes trust (Kosfeld et al., 2005) and/or suspicion (Turner et al., 1999) because in a safe environment one has the privilege of being selective. Thus, we hypothesize that the higher the baseline vasal level of OT in relatively safer environments, the greater the sensitivity to social cues – promotion of proximity to others who are trustworthy and avoidance to those who are not.

Among people with a history of stressful and insecure interactions and a relatively unsafe environment, the need to maintain interpersonal proximity, and thus security, may override considerations that otherwise promote selectivity. Thus, OT is expected to increase affiliation tendencies among people with early life adversity to the point of overlooking other people’s flaws, because in stressful environments being with others, even imperfect others (e.g., unfaithful romantic partners), enhances survival more than being alone.

Vasopressin regulates social-related threat by increasing vigilance and the motivation to act (Bos et al., 2012). Maintaining vigilance is costly and organisms are motivated to regulate vigilant behaviors and activate these behaviors only when necessary (Coan and Sbarra, 2015). These behaviors are enacted when cues in the environment, such as signs of danger or low proximity to others, suggest the possibility of threat (Bowlby, 1973). Individuals with a history of safe interactions with others and an early safe environment are less likely to display vigilant behaviors as these behaviors come at the expense of other important tasks (e.g., exploration and caring). Individuals with no history of childhood adversity, therefore, are expected to show a suppressed AVP response such that AVP will not maintain behavioral vigilance. Individuals with an insecure life history and/or an early unsafe environment are likely to display hypervigilant responses (i.e., better detection of threats), and these responses are expected to be associated with higher AVP.

To examine these predictions, we conducted two studies. In Study 1, we examined the links between people’s basal levels of OT and AVP and hypervigilant responses as a function of early life stress. In Study 2, we examined the effects of OT administration on hypervigilant responses as a function of early life stress.

## Study 1

### Methods

#### Participants

We conducted the study on 40 undergraduates from Bar-Ilan University (24 men and 16 women, aged 18 to 31, *M* = 23.71, *SD* = 3.27), who were individually invited to a lab. We did not include in the study women who were pregnant or breastfeeding, individuals who suffer from psychiatric disorders, head injuries, heart disorders, and addiction to alcohol or drugs, and those who take medication with endocrine-related effects (Gouin et al., 2010). This study was carried out in accordance with the recommendations of APA guidelines and Declaration of Helsinki with written informed consent from all subjects. The study was approved by the Bar-Ilan’s and Interdisciplinary Center (IDC) Herzliya’s IRB. Each participant received 150 NIS (approximately 35$) for her or his participation. The sample size was predetermined by a power analysis (Faul et al., 2009) to allow 80% power for detecting differences between two moderate-sized slopes (the main hypothesis in the current study). The analysis indicated that we need at least 46 participants. We decided to sample only 40 participants because of the kit size in analyzing basal levels of neuropeptides, and therefore, the observed power in Study 1 is 75.15%.

#### Materials and Procedure

Upon arrival to the laboratory, participants signed an informed consent form. Then they had their blood drawn to assess their basal levels of OT and AVP. All blood draws took place in the mid-afternoon and were conducted by a nurse. Blood, from the antecubital-vein, was drawn into chilled Li-Heparin vacutainer tubes containing Aprotinin (Sigma) 500 KIU, per 1 ml blood. Blood samples were then centrifuged at 1,600 × *g* for 20 min at 4°C. Plasma was transferred to plastic tubes and stored at -80°C until assayed.

Determination of hormones was performed by using a 96-plate commercial kits, OT-ELISA kit ADI-900-153 and Arg^8^VP-EIA kit ADI 900-017 (Assay-Design, MI, United States), consistent with previous research (Apter-Levi et al., 2014). The immunoassay for the determination of un-extracted plasma OT was found to be sensitive and reliable in previous studies (Feldman et al., 2011, 2013; Gordon et al., 2010a,b; Schneiderman et al., 2012, 2014; Weisman et al., 2013; Apter-Levi et al., 2014). The kit’s instruction recommended, but did not mandate the use of extraction prior to EIA analysis. For this reason, we randomly selected and compared samples in all our studies (amounting to overall *N* = 673 plasma samples) OT values from extracted and un-extracted samples and found that the two showed very similar results for the current kit. We found that when using the Oxytocin ELISA kit 900-153, the extracted and non-extracted samples gave very similar values. For instance, in the current study, the mean of eight direct un-extracted blood samples was 383.75 and that of 8 extracted blood sample was 374.5 pg/ml. Samples were diluted (1:5) in the suitable assay buffer for OT or AVP and further treated according to kit’s instructions. Dilution has already shown to give results within the linear portion of the standard curve. Measurements performed in duplicates.

Sample Concentration of OT or AVP, were calculated by MatLab-7 (MathWorks, Natick, MA, United States) according to the relevant standard curve. The intra-assay coefficients of variation of OT or AVP were less than 10.3 and 6.09%, respectively.

Following the collection of blood samples, we obtained a self-report of positive and negative events experienced during childhood, and the perceived stress associated with those events, by employing an edited version of the Life Experiences Survey (Sarason et al., 1978) (i.e., LONGSCAN). The survey comprised 30 items relating to events such as “moved to a new place” and “trouble with the law.” For each participant, we calculate the number of negative events experienced during childhood. We also assessed participants’ maltreatment histories by employing the 28-item Childhood Trauma Questionnaire (CTQ-SF) (Bernstein et al., 2003), which comprises five subscales, three assessing abuse (emotional, physical, and sexual) and two assessing neglect (emotional and physical). Each subscale has five items and there is a three-item Minimization-Denial subscale to check for extreme response bias, specifically attempts by respondents to minimize their childhood abuse experiences. Participants are asked to report on the prevalence of each event using a 5-point scale, ranging from *never true* (1) to *very often true* (2). For each participant, we calculate the level of childhood emotional (α = 0.62), physical (α = 0.84), and sexual (α = 0.89) abuse and emotional (α = 0.88) and physical (α = 0.61) neglect by averaging the answers in the relevant items. Next, we conducted a parallel analysis to determine the ideal number of factors for the variables of negative life events and childhood trauma, which recommended one-factor solution. A factor analysis with maximum likelihood as the extraction method and oblimin rotation has revealed that all six factors were significantly loaded on one factor (0.90 for emotional abuse, 0.67 for sexual abuse, 0.57 for physical abuse, 0.46 for emotional neglect, 0.35 for negative life events, and 0.33 for physical neglect), which we named “early life stress” and that had excellent goodness of fit, χ^2^_(9)_ = 9.41, *p* = 0.40. For each participant, we calculated his or her level of early life stress by employing Anderson-Rubin method.

**FIGURE 1:**
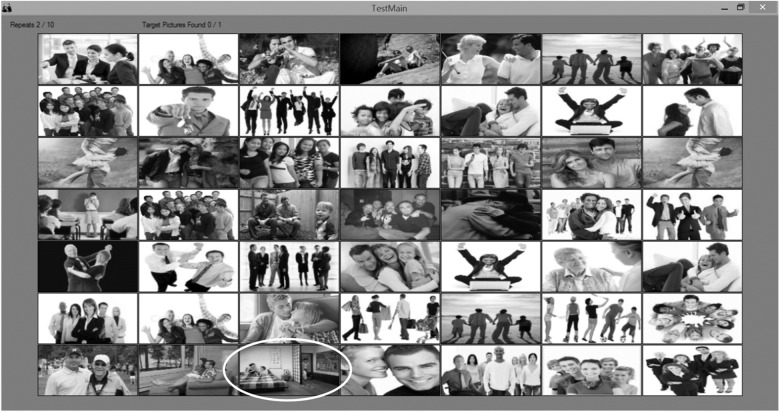
Computerized infidelity detection task. Participants are instructed to click as quickly as possible with the left button of the mouse on the target picture, which portray signs of infidelity. An example of a target picture is marked by a white oval shape.

Following these questionnaires, we assessed participants’ ability to detect social-related (i.e., infidelity) and non-social-related threats (i.e., poisonous animals) by employing Ein-Dor et al. (2015). Specifically, to examine participants’ ability to detect an act of infidelity, we employed a computerized detection task, in which participants were instructed to detect as quickly as possible a picture portraying an act of infidelity (i.e., a target picture) out of a matrix of control pictures depicting neutral social interactions (e.g., a group of people jumping in the air, two people hugging, two people dancing; see Figure 1 for an example of one matrix). The task commenced with three practice trials, in which participants were presented with a matrix of nine pictures (3 × 3: one target picture and eight control pictures). Participants were instructed to click as quickly as possible with the left button of the mouse on the target picture. Upon successful detection of the target picture, a new matrix was presented. Following the practice trials, participants were presented with ten test trials, which comprised matrices of 49 pictures (7 × 7: one target picture and 48 control pictures). The location of the target picture was randomized for each trial. The software coded the time taken to detect the target picture (in milliseconds) and the number of attempts to do so (i.e., number of mouse clicks). For each participant, we calculated the sensitivity in detecting the pictures portraying infidelity by counting the total number of mistakes in performing the task, such that higher scores reflect lower sensitivity.

**FIGURE 2:**
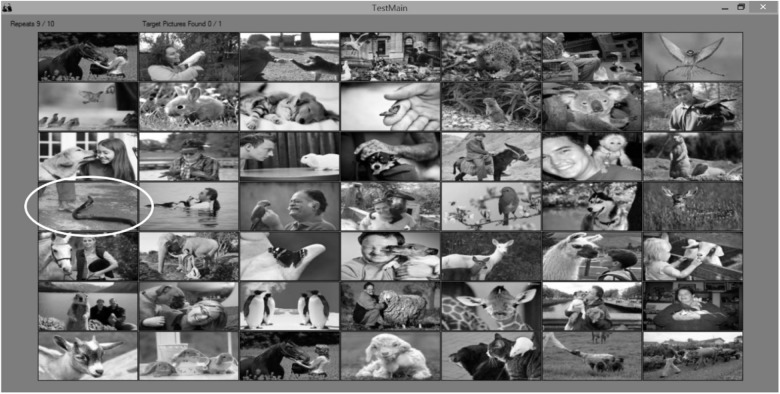
Computerized poisonous animals detection task. Participants are instructed to click as quickly as possible with the left button of the mouse on the target picture, which portray poisonous animals. An example of a target picture is marked by a white oval shape.

To assess participants’ ability to detect poisonous animals as a threat, we employed a second task with similar parameters in which they were asked to detect as quickly as possible a target picture portraying a threat from a poisonous animal (e.g., a person bitten by a snake) out of a matrix of control pictures (e.g., a person with a monkey on his shoulder, a dog licking a woman’s face, a person holding a parrot; see Figure 2 for an example of one matrix). Following these tasks, participants were debriefed and thanked. The software coded the time taken to detect the target picture (in milliseconds) and the number of attempts to do so (i.e., number of mouse clicks). For each participant, we calculated the sensitivity in detecting the pictures portraying a threat from poisonous animal by counting the total number of mistakes in performing the task, such that higher scores reflect lower sensitivity.

### Results and Discussion

As a preliminary analysis, we examine biological sex differences in basal levels of OT and AVP and in early life stress. Research has indicated that males and females differ in OT and AVP basal levels and in the prevalence of OT and AVP receptors in the brain (e.g., Dumais and Veenema, 2016), as well as in the effects of early life stress (e.g., Pechtel and Pizzagalli, 2011). In a series of independent samples *t*-test (see Table 1), we have detected no significant differences (and low effect sizes: *Cohen’s ds* lower or equal to 0.20) between males and females. Because of the documented sexual dimorphism in OT and AVP systems, we adjusted the main analyses for the contribution of biological sex nevertheless. Pattern of associations between main study measures as well as means and standard deviations are presented in Table 2.

**Table 1:** Biological sex differences in OT and AVP basal levels and early life stress (Study 1).

	Male		Female		
			
	*M*	*SD*	*M*	*SD*	*t*_(38)_	*Cohen’s d*
AVP	229.71	283.81	197.66	74.35	-0.44	-0.14
OT	646.32	606.25	622.14	270.78	-0.15	-0.05
Early life stress	0.10	1.14	-0.15	0.74	-0.77	-0.25


**Table 2:** Pattern of associations between main study measures as well as means and standard deviations (Study 1).

		1	2	3	4	5
1	# mistakes infidelity	–
2	# mistakes animals	0.57***	–
3	AVP	-0.11	-0.07	–
4	OT	0.05	-0.09	0.66***	–
5	Early life stress	0.35**	0.07	-0.21	-0.04	–
	Mean	2.49	1.51	216.89	636.65	0.00
	Standard deviation	3.04	1.24	223.34	495.07	1.00


^∗∗^*p* < 0.01, ^∗∗∗^*p* < 0.001.

**FIGURE 3:**
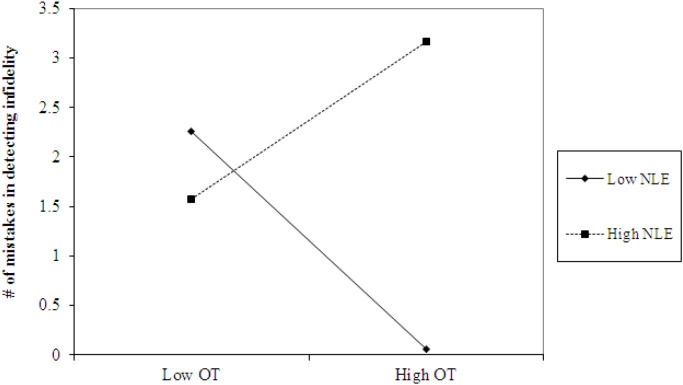
Accuracy in detecting infidelity (number of mistakes) as a function of OT and early life stress (NLE). High OT was linked with lower accuracy (more mistakes) in detecting infidelity among people with high early life stress (i.e., not seeing flaws), but with higher accuracy among people with low early life stress and safer environments (i.e., greater sensitivity to social cues).

Using hierarchical regression analyses, we examined the number of mistakes in detecting infidelity and poisonous animals as a function of participants’ basal levels of OT and AVP, early life stress, and their interactions. We also included gender as a covariate to adjust the analyses for its effect. Regression coefficients are presented in Table 3. The analyses revealed the expected interaction between OT and early life stress in detecting infidelity (see Figure 3) but not poisonous animals. These interactions indicate that early life stress is selectively associated with vigilance to social cues, and is not associated with general vigilance to non-social stimuli. As predicted, simple slopes tests indicated that high OT was linked with lower accuracy (more mistakes) in detecting infidelity among people with high early life stress (one standard deviation above the mean of early life stress) (i.e., not seeing flaws; *b* = 3.49, *p* = 0.022), but with higher accuracy among people with low early life stress and safer environments (one standard deviation below the mean of early life stress) (i.e., greater sensitivity to social cues; *b* = -4.10, *p* = 0.021).

**Table 3:** Regression coefficient for predicting the accuracy in detecting infidelity and poisnious animals by AVP, OT, and early life stress (Study 1).

	# mistakes infidelity		# mistakes animals	
		
	*b*	*β*	*b*	*β*
AVP	-0.39	-0.13	0.01	0.01
OT	0.45	0.15	-0.12	-0.09
Early life stress	0.99*	0.32	0.09	0.06
Biological sex	-0.85	-0.14	0.06	0.02
AVP × Early life stress	-4.34**	-0.39	-1.90*	-0.29
OT × Early life stress	3.79*	0.36	1.86	0.20


^∗^*p* < 0.05, ^∗∗^*p* < 0.01.

**FIGURE 4:**
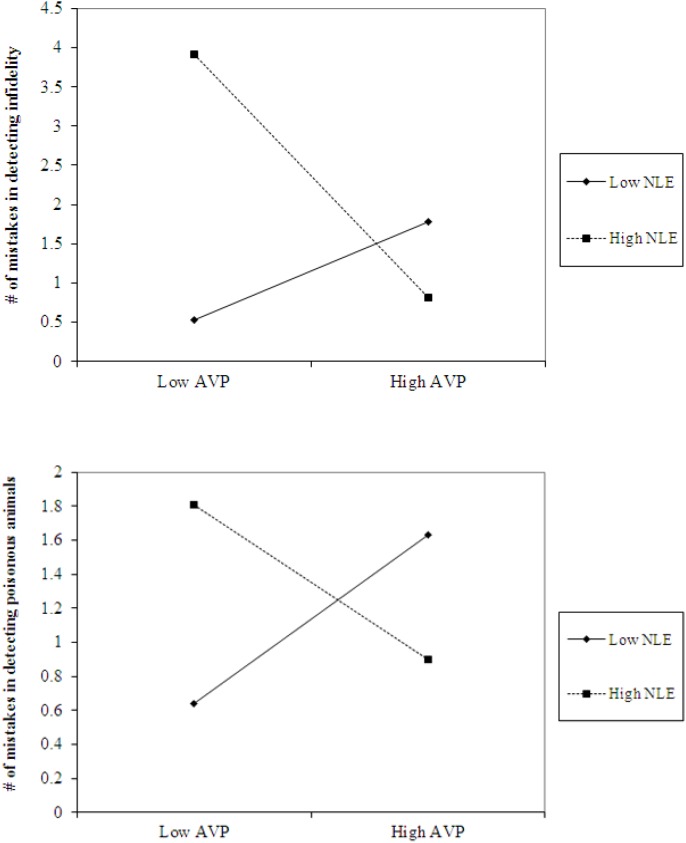
Accuracy in detecting infidelity (top panel) and poisonous animals (bottom panel) as a function of AVP and early life stress (NLE). High AVP was linked with higher accuracy in detecting infidelity (i.e., fewer mistakes) and poisonous animals among people with high early life stress (i.e., better ability to detect various threats) but with lower accuracy among people with low early life stress and, thus, safer environment (i.e., lower vigilance to threats).

The analyses also revealed the expected interaction between AVP and early life stress in detecting infidelity (see Figure 4, top panel) and poisonous animals (see Figure 4, bottom panel). As predicted, we found that high AVP was linked with higher accuracy in detecting infidelity (i.e., fewer mistakes; *b* = -5.26, *p* = 0.034) and poisonous animals (*b* = -1.72, *p* = 0.05) among people with high early life stress (one standard deviation above the mean of early life stress) (i.e., better ability to detect various threats) but with lower accuracy (*b* = 3.42, *p* = 0.03 for infidelity and *b* = 1.77, *p* = 0.04 for animals) among people with low early life stress and, thus, safer environments (one standard deviation below the mean of early life stress) (i.e., lower vigilance to threats). Overall, the models explained 34.1% of the variance in accuracy in detecting infidelity, and 14.8% of the variance in accuracy in detecting poisonous animals.

As predicted, early life stress qualified the function of OT and AVP in an evolutionary predictable way; under conditions of high early life stress, AVP promoted high vigilance to social and non-social threats, while OT safeguarded people’s social network by lowering their ability to detect social flaws (but not other non-social threats). Conversely, under conditions of low early life stress and safer environments, AVP promoted the maintenance of lower vigilance to social and non-social threats, and, thus, the preservation of metabolic energy, while OT promoted the accurate appraisal of people’s social network by increasing the perception and detection of social cues. Although Study 1 supported our main predictions it is correlational in nature. In Study 2, we wished to replicate part of the results of Study 1 by examining the causal link between OT and hypervigilant responses as a function of early life stress.

## Study 2

In Study 2, we examined whether early life stress moderated the effects of OT administration on hypervigilant responses. To do so, we examined people’s ability to detect social (i.e., infidelity) and non-social (i.e., poisonous animals) threats before and after an OT administration (OT vs. Placebo). We predicted that for people with a history of secure interactions and a relatively safe environment, OT administration will improve the ability to detect a social threat but not a non-social threat. Conversely, for people with a history of insecure interactions and a relatively unsafe environment, OT administration will decrease the ability to detect a social threat but not a non-social threat. We did not use AVP administration because it was not available in Israel during the time of the study (2016).

### Methods

#### Participants

Eighty-four Israeli undergraduates (42 women and 42 men, aged 19 to 48, *M* = 24.23, *SD* = 3.51) from the Interdisciplinary Center (IDC) Herzliya, participated in the study for a monetary compensation of 150 NIS (approximately 35$). The criteria for exclusion and inclusion were similar to those of Study 1. The study was approved by the Bar-Ilan’s and Interdisciplinary Center (IDC) Herzliya’s IRBs. The sample size was predetermined by a power analysis (Faul et al., 2009) to allow 80% power for detecting a within-between-subject interaction (the hypothesized effect in the current study) of at least 2.5% explained variance (a weak-to-moderate effect).

#### Materials and Procedure

The experimenter contacted the participants by phone to schedule the date for the laboratory meeting and sent them a Qualtrics link by mail with packet of questionnaires to fill prior their arriving to the laboratory (they were asked to fill it at least 24 h before). The packet included a measure of stressful life events (LES; Sarason et al., 1978) and childhood trauma (CTQ-SF; Bernstein et al., 2003) for appraising early life stress (α = 0.86), as in Study 1. Then, participants were individually invited to the laboratory and upon their arrival, they were asked to complete the computerized infidelity detection task and the poisonous animals detection task, as in Study 1.

After completing these tasks, participants were randomly assigned to experimental conditions. Participants were self-administered 16 IU of synthetic oxytocin [Syntocinon (Novartis), imported from Switzerland] delivered intranasally in two puffs per nostril (with 4 IU per puff), or a matching placebo nasal spray containing non-active ingredients. This dosage was found to have similar affects as 24 IU (Van IJzendoorn et al., 2012) which is the most frequently used dosage for OT administration studies (78% of all studies reviewed used 20–24 IU; MacDonald et al., 2011). We followed Guastella et al. (2013) recommendations for the standardization of oxytocin nasal administration (i.e., instructions to participants, nasal spray handling, etc.). After the administration, participants were asked to wait 45 min to ensure that the OT levels in the central nervous system had reached a plateau (MacDonald et al., 2011). While doing so they watched a neutral video clip to ensure equal settings for all participants, and to avoid distractions and arousal (video may be reached here^1^).

Approximately 45 min after the administration, participants completed the computerized infidelity detection task and the poisonous animals detection task for a second time (the tasks use randomized stimuli and different picture matrices were, therefore, presented before and after the administration). Finally, participants completed a socio-demographic questionnaire, debriefed and thanked.

### Results and Discussion

Pattern of associations between main study measures as well as means and standard deviations are presented in Table 4. Using independent samples *t*-tests, we found that before the OT administration, the number of mistakes in detecting infidelity [*t*_(82)_ = -0.63, *p* = 0.53] and/or poisonous animals [*t*_(82)_ = -0.70, *p* = 0.49] did not differ significantly between the OT and the placebo groups. This finding establishes the random selection of participants to experimental conditions.

**Table 4:** Pattern of associations between main study measures as well as means and standard deviations (Study 2).

		1	2	3	4	5
1	r# mistakes infidelity before	–
2	r# mistakes animals before	0.39***	–
3	r# mistakes infidelity after	0.50***	0.38***	–
4	r# mistakes animals after	0.34***	0.34**	0.39***	–
5	Early life stress	-0.24*	-0.09	-0.21*	0.02	–
	Mean	41.75	41.64	42.85	42.40	0.00
	Standard deviation	23.96	20.75	20.80	20.49	1.00


*^∗^*p* < 0.01, ^∗∗^*p* < 0.01, ^∗∗∗^*p* < 0.001. *r*# = ranked.*

**FIGURE 5:**
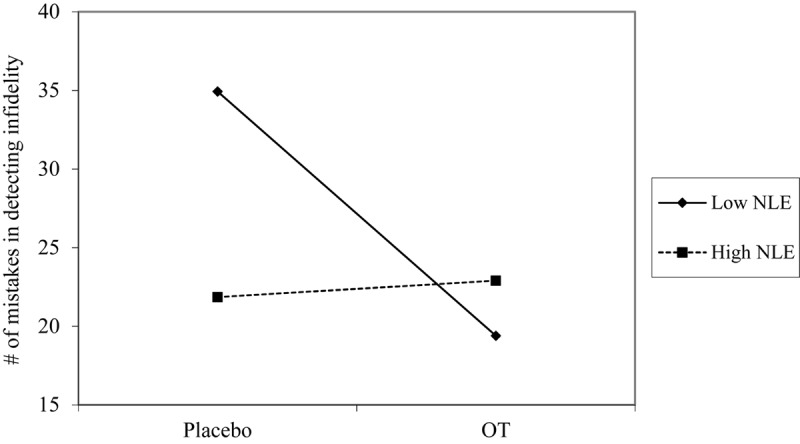
Accuracy in detecting infidelity (number of mistakes) as a function of OT administration and early life stress (NLE). High OT was not linked with accuracy among people with high early life stress (i.e., not seeing flaws), but was linked with higher accuracy among people with low early life stress and safer environments (i.e., greater sensitivity to social cues).

Using hierarchical regression analyses, we examined change in the number of mistakes in detecting infidelity and poisonous animals as a function of OT administration, early life stress, and their interactions. As in Study 1, we included gender as a covariate to adjust the analyses for its effect. Specifically, in the first step of the analyses, we introduced the number of mistakes before the administration, and gender as a covariate. This step allowed us to estimate whether OT administration as a function of early life stress predict the increase and/or decrease in the accuracy in detection. In the second step of the analyses, we added early life stress and OT group (OT = 0.5, placebo = -0.5) as predictors. In the third step of the analyses, we added the interaction between OT group and early life stress. Regression coefficients are presented in Table 5. Because Bonferroni outlier test have revealed the presence of outliers (based on student residuals higher than 3.5) and Breusch-Pagan Test indicated the presence of nonconstant error variance, χ^2^_(1)_ = 253.32, *p* < 0.0001, we used ranked score for the number of mistakes in detection. The analyses revealed the expected interaction between OT and early life stress in detecting infidelity (see Figure 5) but not poisonous animals. This interaction indicates that early life stress is selectively associated with vigilance to social cues, and is not associated with general vigilance to non-social stimuli as in Study 1. As predicted, simple slopes tests revealed that OT administration significantly increased the accuracy among people with low early life stress and safer environments (one standard deviation below the mean of early life stress) (i.e., greater sensitivity to social cues as indicated by fewer mistakes; *b* = -13.51, *p* = 0.009). Conversely and unlike Study 1, for people with high early life stress (one standard deviation above the mean of early life stress), OT administration had no effect on the change in accuracy in detecting a social threat (*b* = 1.21, *p* = 0.836). Thus, unlike high basal levels of OT that were linked with lower sensitivity among people with high early life stress in Study 1, OT administration, which temporarily increases OT levels, did not have similar effect on sensitivity to social cues.

**Table 5:** Regression coefficient for predicting the change in accuracy in detecting infidelity and poisnious animals by OT administration and early life stress (Study 2).

	# mistakes infidelity after		# mistakes animals after	
		
	*b*	β	*b*	β
# of mistakes before	0.45***	0.52	0.34	0.38***
Biological sex	0.25	0.01	-3.43	-0.08
OT administration	-7.25	-0.18	0.22	0.01
Early life stress	-2.69	-0.13	1.96	0.10
OT × Early life stress	8.26*	0.20	-5.83	-0.14


^∗^*p* < 0.05, ^∗∗∗^*p* < 0.001.

## General Discussion

In the current research, we set out to explore whether early life stress qualified the function of OT and AVP in an evolutionary predictable way in keeping with various differential susceptibility models (Belsky, 1997, 2005; Boyce and Ellis, 2005; Del Giudice, 2016). In Study 1, we found that under conditions of high early life stress, AVP promoted high vigilance to social and non-social threats, while OT safeguarded people’s social network by lowering their ability to detect social flaws (but not other non-social threats). This result is in keeping with the strategic pluralism theory (Gangestad and Simpson, 2000), which claim that biparental care is essential to survival in harsh environments, and so early life stress would promote less promiscuous behavior (opposing the developmental-attachment theory suggested by Belsky et al., 1991); and with Schmitt’s (2005) findings regarding sociosexuality in 48 nations, which show that high levels of early life stress are related with less promiscuous behavior and with more stable long term romantic relationships. Conversely, under conditions of low early life stress and safer environments, AVP promoted the maintenance of lower vigilance to social and non-social threats, and, thus, the preservation of metabolic energy, while OT promoted the accurate appraisal of people’s social network by increasing the perception and detection of social cues. In Study 2, we replicated the latter findings and found that OT administration also promoted the accurate appraisal of people’s social network only among people with history of low early life stress and safer environments.

These findings highlight the possibility that via epigenetic processes, neuropeptides such as OT and AVP adapt their function to maximize organisms’ likelihood to survive and reproduce. Other research has already shown that proxies of early life stress (such as attachment styles) are linked with different epigenetic profiles (e.g., Bockmühl et al., 2015; Puglia et al., 2015; Gouin et al., 2017; Ein-Dor et al., 2018) and specifically with the oxytocin receptor gene. The results of the current research suggest that levels of neuropeptides alone do not modulate the behavioral response. Rather, behaviors and cognitions are a function of both levels of OT and AVP and early life experiences. These findings make evolutionary sense because levels of neuropeptides vacillate due to various life circumstances (e.g., delivery and lactation), and if responses to threat were based on levels alone, organisms’ threat response would be maladaptive when there is an incongruency with environmental demands.

The current research also highlights possible differences between OT levels (as was captured in Study 1) and responsiveness (as tested in Study 2). Specifically, in Study 1, basal levels of OT were linked with higher sensitivity in detecting social threats among people with low early life stress, but with lower sensitivity in detecting such threats among people with high early life stress. In Study 2, OT administration was only linked with increased sensitivity in detecting social threats among people with low early life stress (i.e., decrease in the number of mistakes in detection after the administration), and not with decreased sensitivity in detecting such threats among people with high early life stress (i.e., the performance of people with high early life stress was not related to the OT administration). Therefore, it might be that early life stress not only influences the function of various neuropeptides but also their plasticity and system responsiveness. High early life stress might decrease the responsiveness of such systems, as previous research indicates (e.g., Hsiao et al., 2016).

The results of this research notwithstanding, there are some limitations that need to be considered. First, Dick et al. (2015) stated that the choice of environment in gene-environment interaction research is critical. Specifically, they noted that “almost any form of adversity or challenge, at any time in a person’s life, has been used as an alternative index of ‘stress’.” (p. 44) in gene-environment interaction research. In the current research, we chose to concentrate on early life stress as opposed to stress in later stages of development. We did so in keeping with research showing that biological susceptibility is more plausible in early development and less in later development (Del Giudice, 2016). Future research may benefit from exploring the effect of stress in later stages as well as the effects of acute and stressful events as opposed to chronic stress over years. Second, this research is based on studies with a limited sample size and, hence, future studies are needed to replicate the current results. Third, although we examined the basal levels and the administration effects of OT on sensitivity in detecting various threats, we have not examined the effects of AVP administration and the responsiveness of the AVP-related processes. In addition, research has noted that nasal administration of neuropeptides (OT and AVP) is not effective because only 0.005% of the intranasally injected peptide reached the cerebrospinal fluid (CSF) within 1 h of the administration (Leng and Ludwig, 2016). Nevertheless, studies do show that because enormous amounts of peptide are nasally administrated, the levels within the CSF are enough (3 times the natural levels, on average) for behavioral change (Leng and Ludwig, 2016). Fourth, in the current research we did not account for individual differences in the genetic and/or epigenetics profiles, which might play a crucial role in the influence of early life stress on the function of OT and AVP. Finally, basal levels of OT and AVP were estimated by a blood draw that was taken upon arrival to the laboratory. Because a blood draw may be stressful by itself and/or because a visit to a laboratory might be stressing, the OT and AVP levels might be affected and not be baseline levels *per se*. Despite the limitations, our findings provide a glimpse in to the way the environment tailors our bodily functions to optimize survival.

## Author Contributions

AP-P was a Ph.D. student under the supervision of TE-D and RF. AP-P conducted the study and written the method sections. TE-D supervised the work, conducted the analyses, and wrote the first draft. GH and SBB edited the manuscript and wrote the final version of the manuscript. RF supervised the work and with the assistance of OZ-S analyzed the basal levels of OT and AVP.

## Conflict of Interest Statement

The authors declare that the research was conducted in the absence of any commercial or financial relationships that could be construed as a potential conflict of interest.

## Footnotes

1 https://www.youtube.com/watch?v=lu4rtOtkzPE
